# Genetic Diversity of *Campylobacter jejuni* Isolated From Avian and Human Sources in Egypt

**DOI:** 10.3389/fmicb.2019.02353

**Published:** 2019-10-18

**Authors:** Marwa I. Abd El-Hamid, Norhan K. Abd El-Aziz, Mohamed Samir, El-sayed Y. El-Naenaeey, Etab M. Abo Remela, Rasha A. Mosbah, Mahmoud M. Bendary

**Affiliations:** ^1^Department of Microbiology, Faculty of Veterinary Medicine, Zagazig University, Zagazig, Egypt; ^2^Department of Zoonoses, Faculty of Veterinary Medicine, Zagazig University, Zagazig, Egypt; ^3^Department of Bacteriology, Mycology and Immunology, Faculty of Veterinary Medicine, Kafrelsheikh University, Kafrelsheikh, Egypt; ^4^Department of Biology, College of Science, Taibah University, Medina, Saudi Arabia; ^5^Fellow Pharmacist at Zagazig University Hospital, Zagazig, Egypt; ^6^Department of Microbiology and Immunology, Faculty of Pharmacy, Port Said University, Port Said, Egypt

**Keywords:** alleles, *C. jejuni*, *flaA* typing, humans, poultry, virulence

## Abstract

*Campylobacter jejuni* (*C. jejuni*) are able to colonise and infect domestic poultry and also pose a risk for humans. The aim of this study was to determine the extent of genotypic diversity among *C. jejuni* isolates recovered from avian and human sources in Egypt. Furthermore, the short variable region (SVR) of flagellin A (*flaA*) gene was analysed for the presence of allelic variants. Our results showed that *C. jejuni* isolates differ in their capacity to harbour each of the virulence genes alone or when present in various combinations. The *flaA* gene was detected in all *C. jejuni* strains and none of the strains had all the studied virulence genes together. When considering *C. jejuni* strains from the investigated sources, the *cdtC* gene was the most similar, while the *cdtB* and *iam* genes were the most dissimilar. We could identify 13 novel alleles in the analysed strains. The analyses of virulence gene patterns, *flaA* gene sequences and allelic variants showed that *C. jejuni* strains from different sources overlapped largely suggesting potential involvement of poultry in transmitting *C. jejuni* to humans. We also found that the strains isolated from the same host were highly heterogeneous, with chicken strains exhibiting the highest diversity. Moreover, the human strains were clustered closer to chicken ones than to those from pigeon. The results of this study should be taken into consideration when assessing the epidemiology and risk potential of Egyptian *C. jejuni* not only in poultry, but also in humans.

## Introduction

*Campylobacter jejuni* (*C. jejuni*) is one of the most frequent bacterial causes of foodborne gastroenteritis worldwide (European Food Safety Authority, 2014). It has been isolated from chickens, turkeys, pigeons and quails (Vazquez et al., 2010; Kovacic et al., 2013; Ramees et al., 2015), where they colonise the intestinal tract and could contaminate the carcasses during processing (e.g., defeathering and evisceration) (Hermans et al., 2011). Human infection occurs commonly through consumption of contaminated poultry meat (Humphrey et al., 2007).

Many studies addressed the virulence characteristics of *C. jejuni* (Frazão et al., 2017; Maansi et al., 2018). In Egypt, limited studies have focussed on the genotypic diversity (Ahmed et al., 2015) and the antimicrobial resistance of *C. jejuni* (Abd El-Tawab et al., 2018), yet none of these studies explored the virulence patterns of *C. jejuni* among different sources, particularly pigeons.

It is well established that *C. jejuni* exhibits high diversity with regard to the presence of virulence and/or pathogenicity traits including adherence (Coote et al., 2007), invasion (Zheng et al., 2006), toxicity (Abuoun et al., 2005), and molecular mimicry (Datta et al., 2003). Determining the extent of genetic heterogeneity of *C. jejuni* will tell about the disease burden in certain populations and will aid in predicting the potential source of infection (McCarthy et al., 2007); all are valuable information that can be delivered to surveillance and control programmes with an overarching goal of reducing the disease in poultry and its transmission to humans.

The genomic instability of the flagellin gene, due to frequent occurrence of genomic recombination (Harrington et al., 1997), makes it a good candidate for studying the genetic diversity of *C. jejuni.* Therefore, molecular typing of *flaA* gene, and in particular its short variable region (SVR; ∼150 base pairs), has been widely used in studying the genotypic diversity of such bacteria (Meinersmann et al., 1997, 2005; Wardak and Jagielski, 2009). *flaA* typing represents a convenient and cost-effective typing-scheme, which suits the situation in developing countries, particularly when quick characterisation of the strains is needed. Previous studies have demonstrated that direct sequencing of PCR-amplified *flaA*-SVRs is useful for *Campylobacter* genotyping, particularly in short-term and localised epidemiological investigations, allowing similar or higher discriminatory power than multilocus sequence typing (MLST). Furthermore, MLST is unable to distinguish closely related strains in small-scale outbreak investigations and additional methods like *flaA* typing may be required in order to obtain sufficient resolution (Meinersmann et al., 1997; Sails et al., 2003).

*Campylobacter jejuni* isolated from humans and poultry are known to be genetically diverse (Ramonaite et al., 2017). Although *C. jejuni* in Egypt is widely distributed in avian hosts (Rahimi and Ameri, 2011; Abd El-Tawab et al., 2018) and humans (Sainato et al., 2018), there are limited studies that addressed the genetic heterogeneity among Egyptian *C. jejuni*.

To unravel the extent of genetic diversity of Egyptian *C. jejuni*, we analysed both virulence gene profiles and *flaA* allelic variants in *C. jejuni* isolated from chickens, pigeons and humans. The knowledge gained from this study is highly relevant to the epidemiology and control efforts geared toward reducing *C. jejuni* infection in Egypt.

## Materials and Methods

### Samples

The study was conducted during the period from 2015–2018. A total of 270 samples were collected from individual broiler chickens (*n* = 90) [chicken meat (*n* = 65) and cloacal swabs (*n* = 25)] and fresh pigeon droplets (*n* = 180) of different ages from 10 retail outlets in Zagazig city, Sharkia Governorate, Egypt. Moreover, 270 human stool samples were collected from gastroenteritis patients attending Zagazig University hospital located in the same city, Zagazig. The collected samples were transported in an ice box within 3 h to the laboratory for bacteriological analysis. The animal study was approved by the committee of Animal Welfare and Research Ethics, Faculty of Veterinary Medicine, Zagazig University. Regarding the human study, it was approved by the research ethical committee of Faculty of Medicine, Zagazig University and the work was carried out in accordance with the Code of Ethics of the World Medical Association (Declaration of Helsinki) for studies involving humans. Written informed consents were obtained from the patients participating in the research study after a full description of the study purpose.

### Isolation and Identification of *C. jejuni*

For the isolation of *Campylobacter* species, samples were enriched in Preston *Campylobacter* selective enrichment broth (Oxoid, United Kingdom) at 42°C for 48 h. The enrichment cultures were first streaked onto modified charcoal cefoperazone deoxycholate agar (mCCDA; Oxoid, United Kingdom), then transferred onto Columbia agar (Oxoid, United Kingdom) plates supplemented with 5% sterile defibrinated horse blood. The plates were incubated at 42°C in darkness for 48 h under microaerobic conditions (Vandepitte et al., 2003). The presumptive isolates were confirmed as *C. jejuni* by biochemical tests including catalase, oxidase, hippurate and indoxyl acetate hydrolyses, and susceptibility to cephalothin and nalidixic acid (30 μg/disc, each) (Quinn et al., 1994). Genomic DNA was extracted from fresh *C. jejuni* cultures using the QIAamp DNA Mini kit (Qiagen, Germany) according to the manufacturer’s instructions. Polymerase chain reaction (PCR)-based amplification of 23S rRNA and *hipO* genes was done to confirm the identification of *C. jejuni* isolates (Wang et al., 2002).

### PCR-Based Detection of Virulence Genes

*Campylobacter jejuni* isolates were screened for the presence of 7 virulence genes namely *flaA*, *virB11*, *wlaN*, *iam*, *cdtA*, *cdtB* and *cdtC*. All PCR assays were performed in a 25 μL reaction mixture containing 12.5 μL of EmeraldAmp Max PCR Master Mix (Takara, Japan), 1 μL of each primer (20 pM; Metabion, Germany), 6 μL of purified *C. jejuni* DNA and 4.5 μL of nuclease-free water. Primers used for PCR assays are listed in Table 1.

**TABLE 1:** Oligonucleotide primers used for the amplification of genus, species and virulence genes of *C. jejuni* isolates.

**Target gene**	**Primer name**	**Oligonucleotide sequence (5′→3′)**	**Amplicon size (bp)**	**Reference**
23S rRNA	23SF	TATACCGGTAAGGAGTGCTGGAG	650	Wang et al., 2002
	23SR	ATCAATTAACCTTCGAGCACCG		
*hipO*	CJF	ACTTCTTTATTGCTTGCTGC	323	Wang et al., 2002
	CJR	GCCACAACAAGTAAAGAAGC		
*cdtA*	GNW	GGAAATTGGATTTGGGGCTATACT	165	Wieczorek et al., 2012
	IVH	ATCACAAGGATAATGGACAAT		
*cdtB*	VAT2	GTTAAAATCCCCTGCTATCAACCA	495	Wieczorek et al., 2012
	WMI-R	GTTGGCACTTGGAATTTGCAAGGC		
*cdtC*	WMI-F	TGGATGATAGCAGGGGATTTTAAC	555	Wieczorek et al., 2012
	LPF-X	TTGCACATAACCAAAAGGAAG		
*flaA*	FLA242FU	CTATGGATGAGCAATT(AT)AAAAT	402	Meinersmann et al., 1997
	FLA625RU	CAAG(AT)CCTGTTCC(AT)ACTGAAG		
*virB11*	virB-232	TCTTGTGAGTTGCCTTACCCCTTTT	494	Datta et al., 2003
	virB-701	CCTGCGTGTCCTGTGTTATTTACCC		
*wlaN*	wlaN DL-39	TTAAGAGCAAGATATGAAGGTG	672	Kordinas et al., 2005
	wlaN DL-41	CCATTTGAATTGATATTTTTG		
*iam*	IAMF	GCGCAAATATTATCACCC	518	Wieczorek et al., 2012
	IAMR	TTCACGACTACTACTATGCGG		

### *FlaA*-SVR Sequencing and Allelic Typing

Sequencing of internal 402 bp fragments of the *flaA*-SVR amplicons was performed using the previously described primer pair Fla242FU and Fla625RU (Meinersmann et al., 1997). The *flaA* amplicons were purified using the QIAquick PCR purification kit (Qiagen, United States) and sequenced in an ABI 3130 automated DNA Sequencer (Applied Biosystems, United States) using the BigDye^®^ Terminator v3.1 Cycle Sequencing Kit (Applied Biosystems, United States). *flaA-*SVR sequences were compared with those previously published at GenBank using Basic Local Alignment Search Tool (BLAST) available at the National Center for Biotechnology Information (NCBI)^1^. To investigate the genetic relatedness of *C. jejuni* isolates, a phylogenetic tree was built based on *flaA*-SVR sequences using neighbour-joining method (Saitou and Nei, 1987) and the Kimura’s two-parameter method (Kimura, 1980) as an estimator for the evolutionary distance among strains. The topology of the tree was evaluated by 1,000 iterations of a bootstrap analysis. This analysis was done using MEGA software (v. 5) (Tamura et al., 2011). A quantitative assessment of the similarity among the analysed sequences was done by running a Clustal W multiple alignment (Thompson et al., 1994) of all sequences. This enabled the generation of percent identity among pairs of *flaA*-SVR gene sequences without accounting for phylogenetic relationships. This analysis was done using the MegAlign software in the Lasergene (v. 7.1.0) package (DNASTAR, Madison, WI, United States).

To retrieve the allelic variants of *C. jejuni* strains, all nucleotide and peptide sequences of *flaA*-SVRs, using their accession numbers as queries, were used as inputs in *Campylobacter* pubMLST *flaA* Oxford database^2^; these were then compared against reference *C. jejuni* strains deposited in this database. The allelic variants of *flaA-*SVR nucleotides and peptides of *C. jejuni* strains were assigned accordingly.

### Bioinformatics and Data Analyses

PC-ORD Software (v. 5, Oregon, United States), the web-based tools MetaboAnalyst (Chong and Xia, 2018), heatmapper (Babicki et al., 2016) and SPSS software (v. 25, IBM, United States) were used for bioinformatics and statistical analyses of the data. To analyse the overall clustering pattern of *C. jejuni* strains, the frequency of each virulence gene, scored as binary data (present = 1, absent = 0), was used as input in a non-metric multidimensional scaling (nMDS) analysis based on the Euclidean distance. To visualise the association between the studied virulence genes and thus their contribution to strain clustering pattern, we performed a hierarchical clustering using the virulence genes scored as binary data. To determine the relationship between the strain sources, a hierarchical clustering, based on Euclidean distances, was generated using the frequency of the presence of each virulence gene in each source as inputs. The estimated Euclidean distances were used to assess the degree of convergence among strain sources. To visualise the association between the strain sources and the virulence genes, a 2-D correspondence analysis was done, which explained 96% of the variance in the data. A third dimension was not used as its contribution was very low (proportion of inertia = 0.4). To determine the degree of similarity of each virulence gene among strains isolated from various hosts, we applied a random forest non-parametric classification method as described previously (Babicki et al., 2016). The degree of similarity of each gene among *C. jejuni* strains was determined considering the following isolation sources: human, chicken meat, chicken cloacal swabs and pigeon; chicken (meat and cloacal swabs together), human and pigeon; chicken (meat and cloacal swabs together) and human; human and pigeon; human and birds (chicken and pigeon). In this approach, the frequency of each virulence gene was firstly used to build up random forest classification model (the model prediction is based on the majority vote of an ensemble of 500 tree trial; out of bag error, OOB error = 0.6) in the respective source comparison. The degree of similarity of each gene was then evaluated by measuring the increase of the OOB error when the respective gene is permuted. To quantify the discriminatory power of each gene, a classic univariate receiver operating characteristic (ROC) curve was created using inputs similar to that described for the random forest classification. This enabled estimating the area under the curve (AUC) for each gene, which ranges from 0 to 1 (0 = least discriminatory power; 1 = highest discriminatory power). It was only technically possible to run the ROC analysis on the virulence genes comparing human and bird isolates.

### Genotypic Diversity of *C. jejuni* Strains

Both Simpson’s (D_1_) (Simpson, 1949) and Shannon’s (H) (Shannon, 1948) diversity indices were used to assess the diversity of *C. jejuni* strains among various sources and within each source using the virulence genes profile as input data. Simpson’s diversity index was calculated based on the equation: 1-D. In this equation, D = $\text{Σ}\,\frac{n\,\left(n-1\right)}{N\,\left(N-1\right)}$ =, where Σ = summation, *n* = total number of bacterial strains showing a particular gene profile and *N* = total number of bacterial strains in the respective source (e.g., in human strains). Simpson’s index has a range of 0–1, with 0 indicates identical bacterial strains and 1 indicates the highest diverse bacterial strains. Shannon’s diversity index (H) was calculated using the following equation: $H=-\sum_{i=1}^{s}P\,i\,l\,n\,p\,i$, where *pi* is the proportion of bacterial strains expressing a particular gene profile divided by the total number of bacterial strains in the respective source, *In* is the natural log of the *pi*, Σ is the summation, and *s* is the number of species. The concordance of the two indices was determined by calculating the Pearson correlation (*r*). This analysis was done using the Past3 software (v. 3.23), Oslo (Hammer et al., 2001).

### Statistical Analysis

Statistical analysis of the frequencies of virulence genes within *C. jejuni* strains isolated from different hosts was assessed by Fisher’s exact test considering *P*-value < 0.05 as a cut off level. Data was analysed using Stata statistical software version 12 (TX, United States).

### Nucleotide Sequence Accession Numbers

The *flaA-*SVR nucleotide sequences generated in this study have been deposited into the GenBank database with accession numbers KX066127-KX066135, MG677923-MG677934 and MK281494-MK281513.

## Results

### Prevalence of *C. jejuni* in Chickens, Pigeons and Humans

The overall prevalence of *C. jejuni* in avian samples (i.e., chicken and pigeon samples) was 11.11% (30/270). Fourteen out of 90 (15.56%) chicken samples were positive for *C. jejuni*; 10 from 65 chicken meat (15.38%) and 4 from 25 cloacal swabs (16%), while the isolation rate of *C. jejuni* in pigeon droplets was 8.89% (16/180). Moreover, 11 *C. jejuni* were isolated from 270 human stool samples (4.07%). Typical *C. jejuni* colonies were small, shiny, round, and grey on mCCDA agar medium. No haemolysis on Columbia blood agar and positive reactions for catalase, oxidase and hippurate and indoxyl acetate hydrolyses presumptively identified *C. jejuni* isolates. Besides, the analysed isolates were typically sensitive to nalidixic acid and resistant to cephalothin as measured by the inhibition zones around the relevant antibiotic discs. The genus and species identification of the isolates were further confirmed by PCR detection of 23S rRNA and *hipO* genes, respectively.

### Frequency of Virulence Genes in *C. jejuni* Isolates

As depicted in Table 2, the *flaA* gene was present in all analysed *C. jejuni* strains (*n* = 41). The *cdtA, cdtB*, and *cdtC* toxin genes were similarly detected in *C. jejuni* strains (80.49%, each), whereas the three CDT toxin genes were simultaneously found in 58.54% of the analysed strains. Only two subunits of the CDT toxin gene (e.g., *cdtBC*, *cdtAC*, and *cdtAB*) were detected in 3 (7.32%), 3 (7.32%) and 6 (14.63%) of the strains, respectively. Moreover, 3 *C. jejuni* strains (7.32%) harboured the *cdtC* gene only and 2 strains (4.88%) had no CDT toxin genes at all (Supplementary Table S1). Besides, *iam*, *wlaN* and *virB11* genes were found in 34.15, 12.20, and 9.76% of *C. jejuni* strains, respectively.

**TABLE 2:** Occurrence of virulence genes in *C. jejuni* strains under study.

**Source**	**No. of *C. jejuni* strains**	**Frequency of virulence genes, no. (%)**						
		***cdtA***	***cdtB***	***cdtC***	***flaA***	***virB11***	***wlaN***	***iam***
Pigeon	16	14 (87.50)	16 (100.00)	14 (87.50)	16 (100.00)	1 (6.25)	0 (0.00)	8 (50.00)
Chicken	14	9 (64.29)	6 (42.86)	10 (71.43)	14 (100.00)	2 (14.29)	3 (21.43)	6 (42.86)
Human	11	10 (90.91)	11 (100.00)	9 (81.82)	11 (100.00)	1 (9.09)	2 (18.18)	0 (0.00)
Total	41	33 (80.49)	33 (80.49)	33 (80.49)	41 (100.00)	4 (9.76)	5 (12.20)	14 (34.15)
*P-value^∗^*		0.173	0.000^∗^	0.545	1.000	0.763	0.164	0.021^∗^

*^∗^Statistical significance was calculated using Fisher’s exact test (*P*-value < 0.05).*

The presence of the *cdtB* gene in human and pigeon strains was significantly (*P* = 0.0001) higher (100%, each) than in chicken ones (42.86%). Additionally, *iam* gene was significantly (*P* = 0.021) more frequently detected in pigeon strains (50%) when compared to chicken (42.86%) and human ones (0%).

**FIGURE 1:**
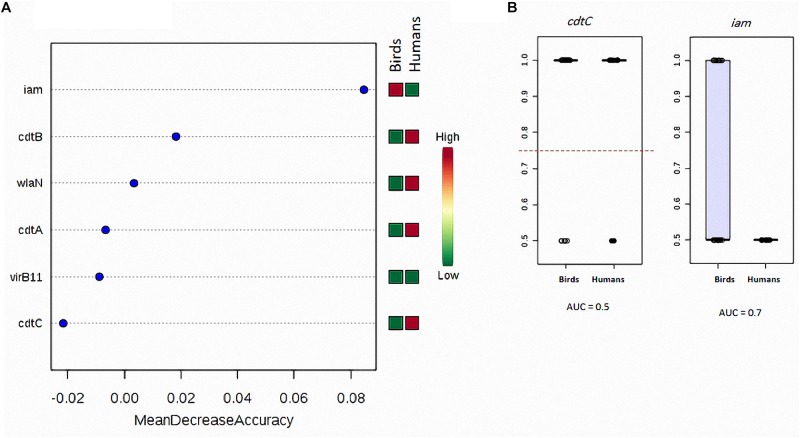
Degree of similarity and discriminatory power of analysed virulence genes in *C. jejuni* strains among various sources. **(A)** Random forest classification showing the predictive accuracy of the studied genes (*X*-axis). The mini heat map shows the frequency distribution of each gene in the respective strain sources. Each dot refers to one gene with the respective mean decrease accuracy. **(B)** Box-plot showing the differences in the distribution of *cdtC* and *iam* genes between birds and human sources of *C. jejuni*. The horizontal dotted line refers to the optimal cutoff value. The AUC data were generated by running the ROC curves as described in the “Materials and Methods” section.

The random forest classification analysis showed that *cdtC* gene exhibited the highest similarity (the least mean decrease accuracy) when considering *C. jejuni* isolated from human and birds (chicken meat, chicken cloacal swabs and pigeon together) (Figure 1A). This gene was also the most similar when considering the following strain sources: (i) human, chicken meat, chicken cloacal swabs and pigeon, (ii) human and chicken (meat and cloacal swabs together), (iii) human and pigeon and (iv) human, chicken and pigeon (Supplementary Figure S1). On contrary, *iam* was the most dissimilar gene when considering *C. jejuni* strains from (i) human and birds (chicken meat, chicken cloacal swabs and pigeon together) and (ii) human and pigeon. The *cdtB* gene was the most dissimilar one when considering *C. jejuni* strains from (i) human, chicken meat, chicken cloacal swabs and pigeon, (ii) human, chicken (meat and cloacal swabs together) and pigeon and (iii) chicken (meat and cloacal swabs together) and humans. ROC-curve analysis (Figure 1B) on the human and bird strains revealed that the *cdtC* gene had lower AUC value (0.5) than that of the *iam* gene (0.7).

The results showed that none of the *C. jejuni* strains harboured all the seven virulence genes together. More than half of the strains (87.80%) possessed three or more virulence genes (Table 3). Irrespective of the strain source, *cdtABC, flaA* combination dominated the overall gene profiles, being encoded by 10 strains (24.39%), followed by *cdtABC, flaA, iam* pattern (7/41, 17.07%) (Supplementary Table S1 and Table 4).

**TABLE 3:** Frequency of *C. jejuni* strains isolated from avian and human sources carrying virulence genes.

**No. of identified virulence genes**	**Human (*n* = 11)**	**Chicken^∗^ (*n* = 14)**	**Pigeon (*n* = 16)**	**Human and pigeon (*n* = 27)**	**Human and chicken^∗^ (*n* = 25)**	**Chicken^∗^ and pigeon (*n* = 30)**	**Human and chicken^∗^ and pigeon (*n* = 41)**
1	0 (0.00)	2 (14.29)	0 (0.00)	0 (0.00)	2 (8.00)	2 (6.67)	2 (4.88)
2	0 (0.00)	3 (21.43)	0 (0.00)	0 (0.00)	3 (12.00)	3 (10.00)	3 (7.32)
3	3 (27.27)	2 (14.29)	3 (18.75)	6 (22.22)	5 (20.00)	5 (16.67)	8 (19.51)
4	5 (45.45)	2 (14.29)	6 (37.50)	11 (40.74)	7 (28.00)	8 (26.67)	13 (31.71)
5	3 (27.27)	2 (14.29)	6 (37.50)	9 (33.33)	5 (20.00)	8 (26.67)	11 (26.83)
6	0 (0.00)	3 (21.43)	1 (6.25)	1 (3.70)	3 (12.00)	4 (13.33)	4 (9.76)
7	0 (0.00)	0 (0.00)	0 (0.00)	0 (0.00)	0 (0.00)	0 (0.00)	0 (0.00)
Simpson diversity	0.64	0.82	0.67	0.6722	0.80	0.82	0.77
Shannon diversity	1.06	1.77	1.223	1.18	1.71	1.7	1.60

*Numbers of strains and percentages in parentheses indicate the proportion of those carrying the respective virulence genes. ^∗^Chicken strains refer to those isolated from chicken meat + chicken cloacal swabs.*

**TABLE 4:** Virulence gene patterns, allelic variants and mutations in *flaA*-SVR of *C. jejuni* isolated from avian and human sources.

**Isolate code no.**	**Source**	**Virulence genes pattern**	***flaA*-SVR nucleotide allele (No. of variations)**	***flaA*-SVR peptide allele (No. of variations)**	**^∗∗^Translation variation reference → clone**	**Accession no.**
1	Pigeon droplets	*cdtABC*, *flaA*, *virB11*, *iam*	**781** (30)	191 (16)	^49^I → ^26^T, ^60^N → ^37^K, ^63^V → ^40^E, ^64^K → ^41^I, ^72^S → ^49^T, ^74^K → ^51^R, ^76^G → ^53^I, ^77^L → ^54^F, ^84^E → ^61^R, ^85^R → ^62^I, ^86^I → ^63^T, ^90^G → ^67^E, ^93^Q → ^70^P, ^94^F → ^71^L, ^95^T → ^72^P, ^96^L → ^73^F	KX066127.1
2	Pigeon droplets	*cdtABC*, *flaA*, *iam*	**731** (32)	191 (16)	^49^I → ^27^T, ^57^A → ^35^E, ^60^N → ^38^K, ^63^V → ^41^E, ^64^K → ^42^I, ^66^T → ^44^I, ^67^I → ^45^M, ^72^S → ^50^T, ^74^K → ^52^R, ^84^E → ^62^R, ^85^R → ^63^I, ^86^I → ^64^T, ^93^Q → ^71^P, ^94^F → ^72^L, ^96^L → ^74^F, ^101^G → ^79^H	KX066128.1
3	Pigeon droplets	*cdtABC*, *flaA*, *iam*	**940**^∗^	58 (10)	^49^I → ^26^T, ^77^L → ^54^V, ^84^G → ^61^A, ^86^I → ^63^S, ^89^S → ^66^R, ^93^Q → ^70^A, ^94^F → ^71^V, ^96^L → ^73^V, ^99^Y → ^76^F, ^101^G → ^78^A	KX066129.1
4	Pigeon droplets	*cdtABC*, *flaA*, *iam*	38 (17)	5 (7)	^49^I → ^27^T, ^71^Q → ^49^H, ^74^K → ^52^N, ^79^R → ^57^C, ^89^S → ^67^F, ^96^L → ^74^F, ^106^K → ^84^P	KX066130.1
5	Pigeon droplets	*cdtABC*, *flaA*, *iam*	**1118** (18)	239 (13)	^56^G → ^32^C, ^57^A → ^33^S, ^59^S → ^35^L, ^62^T → ^38^I, ^71^Q → ^47^I, ^76^G → ^52^A, ^78^T → ^54^K, ^79^R → ^55^C, ^90^G → ^66^T, ^92^V → ^68^E, ^99^Y → ^75^S, ^101^A → ^77^C, ^104^D → ^80^V	KX066131.1
6	Pigeon droplets	*cdtABC*, *flaA*, *iam*	**1275** (19)	209 (13)	^57^A → ^35^S, ^62^T → ^40^I, ^64^K → ^42^I, ^68^G → ^46^A, ^70^T → ^48^S, ^71^Q → ^49^H, ^72^S → ^50^T, ^75^I → ^53^M, ^94^L → ^72^V, ^96^L → ^74^F, ^99^Y → ^77^D, ^102^I → ^80^K, ^104^D → ^82^V	KX066132.1
7	Pigeon droplets	*cdtABC*, *flaA*	**186** (25)	356 (17)	^62^T → ^37^A, ^63^I → ^38^V, ^64^K → ^39^I, ^65^A → ^40^V, ^72^S → ^47^P, ^73^S → ^48^F, ^74^K → ^49^Q, ^78^T → ^53^S, ^85^R → ^60^S, ^86^I → ^61^V, ^88^S → ^63^T, ^93^Q → ^68^S, ^96^L → ^71^P, ^99^Y → ^74^D, ^101^G → ^76^S, ^103^D → ^78^H, ^104^D → ^79^G	KX066134.1
8	Pigeon droplets	*cdtABC*, *flaA*	9 (26)	60 (15)	^33^A → ^8^T, ^49^I → ^24^V, ^52^E → ^27^K, ^57^A → ^32^E, ^66^T → ^41^I, ^67^I → ^42^T, ^72^S → ^47^T, ^77^L → ^52^F, ^82^T → ^57^R, ^83^G → ^58^V, ^90^G → ^65^S, ^94^F → ^69^A, ^96^L → ^71^H, ^102^L → ^77^R, ^106^Q → ^81^L	KX066135.1
9	Pigeon droplets	*cdtAB*, *flaA*	**781** (31)	191 (14)	^49^I → ^26^T, ^57^A → ^34^E, ^60^N → ^37^K, ^63^V → ^40^E, ^64^K → ^41^I, ^72^S → ^49^T, ^74^K → ^51^R, ^76^G → ^53^I, ^84^E → ^61^R, ^85^R → ^62^I, ^86^I → ^63^T, ^93^Q → ^70^P, ^94^F → ^71^L, ^96^L → ^73^F	MK281505
10	Pigeon droplets	*cdtAB*, *flaA, iam*	**1118** (17)	239 (13)	^56^G → ^33^C, ^57^A → ^34^S, ^59^S → ^36^L, ^62^T → ^39^I, ^68^G → ^45^A, ^71^Q → ^48^N, ^76^G → ^53^A, ^78^T → ^55^K, ^79^R → ^56^C, ^90^G → ^67^T, ^92^V → ^69^E, ^101^A → ^78^C, ^104^D → ^81^V	MK281506
11	Pigeon droplets	*cdtBC*, *flaA*	**938** (19)	209 (12)	^57^A → ^34^S, ^62^T → ^39^I, ^64^K → ^41^I, ^68^G → ^45^A, ^70^T → ^47^S, ^71^Q → ^48^N, ^72^S → ^49^T, ^75^I → ^52^M, ^94^L → ^71^V, ^96^L → ^73^F, ^102^I → ^79^K, ^104^D → ^81^V	MK281507
12	Pigeon droplets	*cdtABC*, *flaA*	177 (10)	74 (5)	^32^I → ^38^T, ^36^T → ^42^A, ^58^S → ^64^G, ^71^Q → ^77^L, ^102^I → ^108^L	MK281508
13	Pigeon droplets	*cdtABC*, *flaA*, *iam*	**1486** (11)	92 (8)	^19^Q → ^11^L, ^26^M → ^18^I, ^29^L → ^21^I, ^63^V → ^55^M, ^77^V → ^69^F, ^96^I → ^88^V, ^97^K → ^89^Q, ^99^Y → ^91^F	MK281509
14	Pigeon droplets	*cdtABC*, *flaA*	**756** (26)	58 (9)	^49^I → ^26^T, ^74^K → ^51^N, ^86^I → ^63^S, ^89^S → ^66^R, ^94^F → ^71^V, ^96^L → ^73^V, ^99^Y → ^76^F, ^101^G → ^78^A, ^106^K → ^83^P	MK281510
15	Pigeon droplets	*cdtBC*, *flaA*	38 (18)	239 (5)	^49^I → ^27^T, ^74^K → ^52^N, ^89^S → ^67^R, ^96^L → ^74^F, ^106^Q → ^84^P	MK281511
16	Pigeon droplets	*cdtABC*, *flaA*	**1183**^∗^	239 (15)	^57^A → ^32^S, ^59^S → ^34^L, ^62^T → ^37^I, ^64^K → ^39^I, ^70^T → ^45^S, ^71^Q → ^46^I, ^72^S → ^47^T, ^75^I → ^50^M, ^90^G → ^65^T, ^94^F → ^69^V, ^96^L → ^71^F, ^99^Y → ^74^S, ^101^A → ^76^C, ^102^I → ^77^K, ^104^D → ^79^V	MK281512
17	Chicken meat	*cdtABC*, *flaA*, *wlaN*, *iam*	177 (8)	74 (5)	^32^I → ^38^T, ^36^T → ^42^A, ^58^S → ^64^G, ^71^Q → ^77^L, ^102^I → ^108^L	MG677923.1
18	Chicken meat	*cdtABC*, *flaA*, *virB11*, *iam*	177 (9)	74 (5)	^10^Q → ^10^E, ^23^N → ^23^Y, ^39^N → ^39^D, ^76^G → ^76^D, ^89^S → ^89^L	MG677924.1
19	Chicken meat	*cdtABC*, *flaA*, *iam*	**1486** (11)	92 (7)	^19^Q → ^13^L, ^26^M → ^20^I, ^63^V → ^57^M, ^80^F → ^74^I, ^87^F → ^81^L, ^97^K → ^91^Q, ^104^D → ^98^E	MG677925.1
20	Chicken meat	*cdtAB*, *flaA*, *iam*	177 (12)	74 (8)	^5^A → ^11^V, ^32^I → ^38^T, ^36^T → ^42^A, ^45^S → ^51^N, ^58^S → ^64^G, ^71^Q → ^77^L, ^81^E → ^87^A, ^102^I → ^108^L	MG677926.1
21	Chicken cl. swab	*cdtAB*, *flaA*	**526**^∗^	211 (13)	^62^T → ^38^I, ^67^I → ^43^L, ^71^Q → ^47^H, ^72^S → ^48^T, ^74^K → ^50^I, ^80^F → ^56^V, ^85^K → ^61^S, ^86^I → ^62^V, ^94^F → ^70^A, ^95^T → ^71^A, ^99^Y → ^75^A, ^102^I → ^78^V, ^103^D → ^79^G	KX066133.1
22	Chicken cl. swab	*cdtABC*, *flaA*, *virB11*, *wlaN*	177 (9)	74 (6)	^5^A → ^11^V, ^32^I → ^38^T, ^36^T → ^42^A, ^58^S → ^64^G, ^71^Q → ^77^L, ^102^I ^108^L	MK281494
23	Chicken cl. swab	*cdtAC*, *flaA*, *wlaN*, *iam*	177 (12)	74 (8)	^5^A → ^11^V, ^32^I → ^38^T, ^36^T → ^42^A, ^45^S → ^51^N, ^58^S → ^64^G, ^71^Q → ^77^L, ^81^E → ^87^A, ^102^I → ^108^L	MK281513
24	Chicken cl. swab	*flaA*	**288**^∗^	92 (9)	^10^Q → ^8^E, ^23^N → ^21^Y, ^26^M → ^24^I, ^39^N → ^37^D, ^63^V → ^61^S, ^76^G → ^74^D, ^80^F → ^78^I, ^87^F → ^85^L, ^89^S → ^87^L	MK281495
25	Chicken meat	*cdtC*, *flaA*	**1486** (12)	92 (7)	^19^Q → ^15^L, ^26^M → ^22^I, ^29^L → ^25^I, ^39^N → ^35^D, ^63^V → ^59^M, ^97^K → ^93^Q, ^104^D → ^100^E	MK281496
26	Chicken meat	*flaA*	177 (5)	74 (2)	^39^N → ^15^D, ^80^F → ^56^I	MK281497
27	Chicken meat	*cdtC*, *flaA*	**288** (8)	92 (4)	^26^M → ^21^I, ^63^V → ^58^M, ^87^F → ^82^L, ^104^D → ^99^E	MK281498
28	Chicken meat	*cdtAC*, *flaA*, *iam*	9 (27)	211 (15)	^33^A → ^8^T, ^62^T → ^37^I, ^67^I → ^42^L, ^72^S → ^47^T, ^74^K → ^49^I, ^80^F → ^55^V, ^82^T → ^57^R, ^85^K → ^60^S, ^86^I → ^61^V, ^94^F → ^69^A, ^95^T → ^70^A, ^96^L → ^71^H, ^99^Y → ^74^A, ^102^I → ^77^V, ^103^D → ^78^G	MK281499
29	Chicken meat	*cdtC*, *flaA*	**526** (23)	211 (13)	^49^I → ^24^V, ^67^I → ^42^L, ^71^Q → ^46^H, ^72^S → ^47^T, ^74^K → ^49^I, ^80^F → ^55^V, ^83^G → ^58^V, ^85^K → ^60^S, ^86^I → ^61^V, ^94^F → ^69^A, ^95^T → ^70^A, ^102^I → ^77^V, ^103^D → ^78^G	MK281500
30	Chicken meat	*cdtAC*, *flaA*	**526**^∗^	60 (14)	^52^E → ^28^K, ^62^T → ^38^I, ^66^T → ^42^I, ^67^I → ^43^T, ^71^Q → ^47^H, ^72^S → ^48^T, ^74^K → ^50^I, ^80^F → ^56^L, ^90^G → ^66^S, ^94^F → ^70^A, ^95^T → ^71^A, ^99^Y → ^75^A, ^102^L → ^78^R, ^103^D → ^79^G	MK281501
31	Human stool	*cdtABC*, *flaA*, *virB11*	177 (8)	74 (5)	^32^I → ^38^T, ^36^T → ^42^A, ^58^S → ^64^G, ^71^Q → ^77^L, ^102^I → ^108^L	MG677927.1
32	Human stool	*cdtABC*, *flaA*	239 (10)	9 (8)	^1^K → ^7^R, ^12^L → ^18^S, ^27^E → ^33^D, ^36^T → ^42^S, ^42^Q → ^48^H, ^73^S → ^79^A, ^92^V → ^98^D, ^98^N → ^104^S	MG677928.1
33	Human stool	*cdtABC*, *flaA*, *wlaN*	**288** (11)	92 (7)	^8^D → ^14^A, ^26^M → ^32^I, ^29^L → ^35^V, ^63^V → ^69^M, ^71^Q → ^77^L, ^87^F → ^93^L, ^104^D → ^110^E	MG677929.1
34	Human stool	*cdtABC*, *flaA*	177 (15)	74 (7)	^10^Q → ^10^E, ^23^N → ^23^Y, ^52^E → ^52^Q, ^65^A → ^65^G, ^76^G → ^76^D, ^88^T → ^88^A, ^89^S → ^89^L	MG677930.1
35	Human stool	*cdtABC*, *flaA*, *wlaN*	**1064** (10)	267 (10)	^4^Q → ^10^P, ^17^M → ^23^L, ^31^N → ^37^T, ^38^F → ^44^I, ^50^N → ^56^Y, ^66^T → ^72^I, ^79^R → ^85^I, ^91^V → ^97^A, ^102^I → ^108^L, ^107^Y → ^113^L	MG677931.1
36	Human stool	*cdtABC*, *flaA*	239 (9)	9 (8)	^8^D → ^14^E, ^10^Q → ^16^H, ^19^Q → ^25^K, ^39^N → ^45^Y, ^46^G → ^52^R, ^62^T → ^68^P, ^74^K → ^80^Q, ^106^K → ^112^Q	MG677932.1
37	Human stool	*cdtABC*, *flaA*	177 (14)	74 (9)	^3^T → ^3^I, ^19^Q → ^19^E, ^34^N → ^34^Y, ^52^E → ^52^D, ^70^T → ^70^S, ^77^V → ^77^A, ^96^I → ^96^V, ^101^G → ^101^S, ^106^K → ^106^E	MG677933.1
38	Human stool	*cdtABC*, *flaA*	**288** (7)	92 (5)	^9^G → ^2^V, ^34^N → ^27^S, ^58^S → ^51^T, ^63^V → ^56^M, ^106^K → ^99^R	MG677934.1
39	Human stool	*cdtAB*, *flaA*	177 (8)	74 (5)	^32^I → ^38^T, ^36^T → ^42^A, ^58^S → ^64^G, ^71^Q → ^77^L, ^102^I → ^108^L	MK281502
40	Human stool	*cdtBC*, *flaA*	177 (8)	74 (4)	^32^I → ^38^T, ^36^T → ^42^A, ^58^S → ^64^G, ^71^Q → ^77^L	MK281503
41	Human stool	*cdtAB*, *flaA*	177 (13)	74 (8)	^19^Q → ^12^E, ^34^N → ^27^Y, ^52^E → ^45^D, ^70^T → ^63^S, ^77^V → ^70^A, ^96^I → ^89^V, ^101^G → ^94^S, ^106^K → ^99^E	MK281504

*SVR, short variable region; cl. swab, cloacal swabs; A, alanine; R, arginine; N, asparagine; D, aspartic acid; C, cysteine; E, glutamic acid; Q, glutamine; G, glycine; H, histidine; I, isoleucine; L, leucine; K, lysine; M, methionine; F, phenylalanine; P, proline; S, serine; T, threonine; Y, tyrosine; V, valine. ^∗^ Nucleotide alleles with asterisks have frameshift rather than point mutations. Novel allelic variants identified in this study are shown in bold. ^∗∗^The information to the left of the arrow refer to the identity and the position on the reference strains (deposited on http://pubmlst.org/campylobacter/) and the information to the right of the arrow show the corresponding identity and position on the query strains.*

The strains from pigeon and human sources (*n* = 27) harbored at least 3–6 virulence genes. In contrast, 50% of the chicken strains were positive for up to 3 virulence genes (Table 3). As shown in Supplementary Table S1 and Table 5, *cdtABC, flaA* dominated the gene profiles in human strains (5/11, 45.45%), was not detected at all in chicken strains and was the second frequent pattern in pigeon strains (5/16, 31.25%). While, *cdtABC, flaA, iam* pattern was the most frequent in pigeon (6/16, 37.50%), it was not detected in any of human strains and was found in only one chicken strain (1/14, 7.14%). The *cdtC, flaA* was the dominant pattern in chicken strains (3/14, 21.43%), but none of human or pigeon strains possessed this pattern.

**TABLE 5:** Distribution of virulence associated genes and identified allele numbers (at both nucleotide and peptide levels) among the entire collection of *C. jejuni* strains.

**Isolate source**	**Virulence gene pattern^∗^**	***flaA*-SVR types (alleles)**	
		**Nucleotide**	**Peptide**
Human (*n* = 11)	*cdtABC*, *flaA* (5); *cdtAB*, *flaA* (2); *cdtABC*, *flaA*, *wlaN* (2); *cdtBC*, *flaA* (1); *cdtABC*, *flaA*, *virB11* (1)	177 (6); 288 (2); 239 (2); 1064 (1)	74 (6); 92 (2); 9 (2); 267 (1)
Chicken (*n* = 14)	*cdtC*, *flaA* (3); *flaA* (2); *cdtABC*, *flaA*, *iam* (1); *cdtABC*, *flaA*, *virB11*, *iam* (1); *cdtAB*, *flaA* (1); *cdtAB*, *flaA, iam* (1); *cdtABC*, *flaA*, *wlaN*, *iam* (1); *cdtABC*, *flaA*, *virB11*, *wlaN* (1); *cdtAC*, *flaA*, *wlaN*, *iam* (1); *cdtAC*, *flaA*, *iam* (1); *cdtAC*, *flaA* (1)	177 (6); 526 (3); 288 (2); 1486 (2); 9 (1)	74 (6); 92 (4); 211 (3); 60 (1)
Pigeon (*n* = 16)	*cdtABC*, *flaA*, *iam* (6); *cdtABC*, *flaA* (5); *cdtBC*, *flaA* (2); *cdtABC*, *flaA*, *virB11*, *iam* (1); *cdtAB*, *flaA* (1); *cdtAB*, *flaA, iam* (1)	781 (2); 38 (2); 1118 (2); 731 (1); 940 (1); 1275 (1); 186 (1); 9 (1); 938 (1); 177 (1); 1486 (1); 756 (1); 1183 (1)	239 (4); 191 (3); 58 (2); 209 (2); 5 (1); 356 (1); 60 (1); 74 (1); 92 (1)

*SVR, short variable region. ^∗^Numbers in parentheses indicate the number of strains containing the respective virulence genes combination.*

### Clustering Pattern of the Virulence Genes and Their Association With *C. jejuni* Strains

**FIGURE 2:**
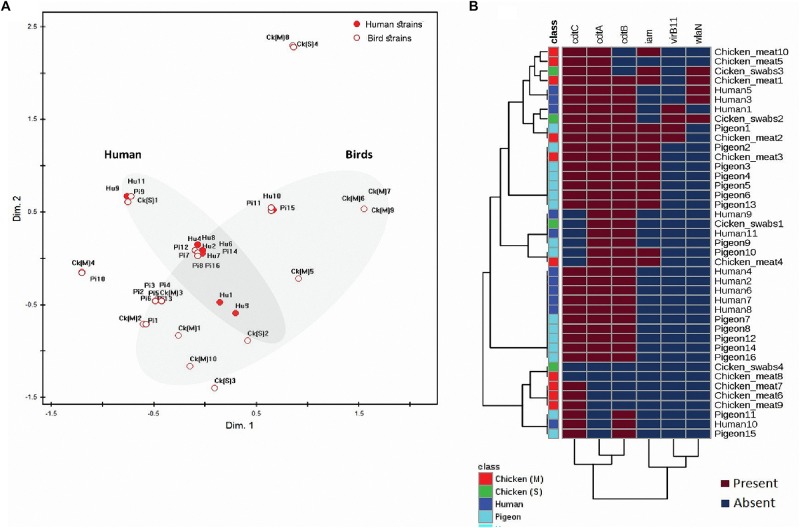
Distribution and clustering of *C. jejuni* strains and the encoded virulence genes. **(A)** Non-metric multidimensional scaling (nMDS) showing the overlap between the bacterial strains (*n* = 41) represented by their sources. **(B)** Hierarchical clustering analyses showing the clustering pattern of both bacterial strains (*n* = 41) and the associated virulence genes (*n* = 6). *FlaA* gene was excluded from this analysis as it was expressed in all strains. The nMDS and the heat map were generated based on the expression profile of the studied virulence genes (*cdtC, cdtA, cdtB, iam, virB11*, and *wlaN*) in all strains (*n* = 41). Hu, human; pi, pigeon; ck (M), chicken meat and ck (S), chicken swabs. Bird strains refer to all strains from chicken and pigeon.

**FIGURE 3:**
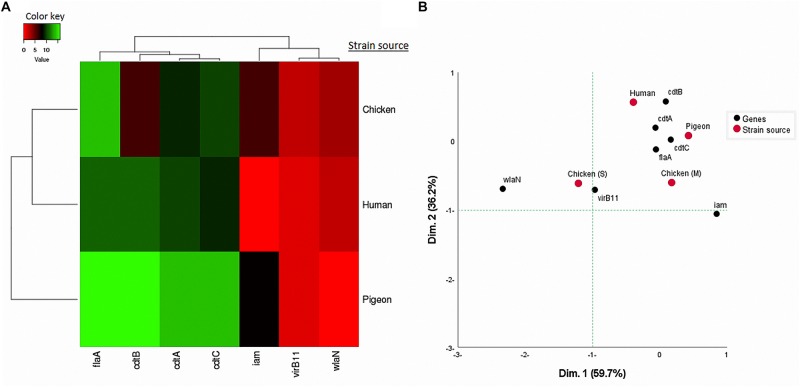
**(A)** Hierarchical clustering of *C. jejuni* strain sources (human, pigeon, and chicken) and the associated virulence genes based on Euclidian distance. **(B)** Two-dimensional correspondence analysis (CA) showing the association between the sources of *C. jejuni* strains [human, pigeon, chicken meat (M) and cloacal swabs (S)] and the studied virulence genes.

Considering the 41 *C. jejuni* strains separately (Figure 2B) and when grouped by their sources (Figure 3A), the virulence genes formed two separate clusters. The first cluster contained *cdtA*, *cdtB*, and *cdtC* genes and the second one included *iam, virB11* and *wlaN* genes. The *flaA* gene was not included in this analysis as it was detected in 100% of the strains. By running a corresponding analysis (Figure 3B), it was evident that *cdtA, cdtB, cdtC*, and *flaA* genes were more associated with *C. jejuni* strains from humans, chicken meat and pigeons. The remaining genes (i.e., *iam, virB11* and *wlaN*) showed a scattered pattern, being more associated with the chicken swab strains.

### Genetic Diversity of *C. jejuni* Strains Based on Their Virulence Genes Profiles

The nMDS and the dendogram analyses (Figures 2, 3A) of *C. jejuni* strains (*n* = 41) showed a non-consistent clustering pattern and high diversity among the analysed strains. The human and bird strains overlapped largely. A similar overlap was observed when analysing the 41 strains grouped by their sources (Figure 3A). The dendogram analysis showed that the human strains were genetically closer to those from chickens (Euclidean distance = 0.19) than to those from pigeons (Euclidean distance = 0.26) (Figure 3A). As shown in Table 3, irrespective of the strain source, the overall Simpson’s and Shannon’s diversity indices of all strains were 0.77 and 1.60, respectively. The highest diversity was observed in *C. jejuni* strains isolated from chickens, followed by those from pigeons and humans. Overall, the results of both diversity indices were highly positively correlated in a significant manner (Pearson correlation, *r* = 0.9; *P* = 0.001).

### Genetic Diversity of *C. jejuni* Based on *flaA-*SVR Gene Sequences

Using the BLAST tool, it was found that our *flaA*-SVR sequences and the flanking regions had 92–99% similarity with those of the previously published *C. jejuni* strains.

**FIGURE 4:**
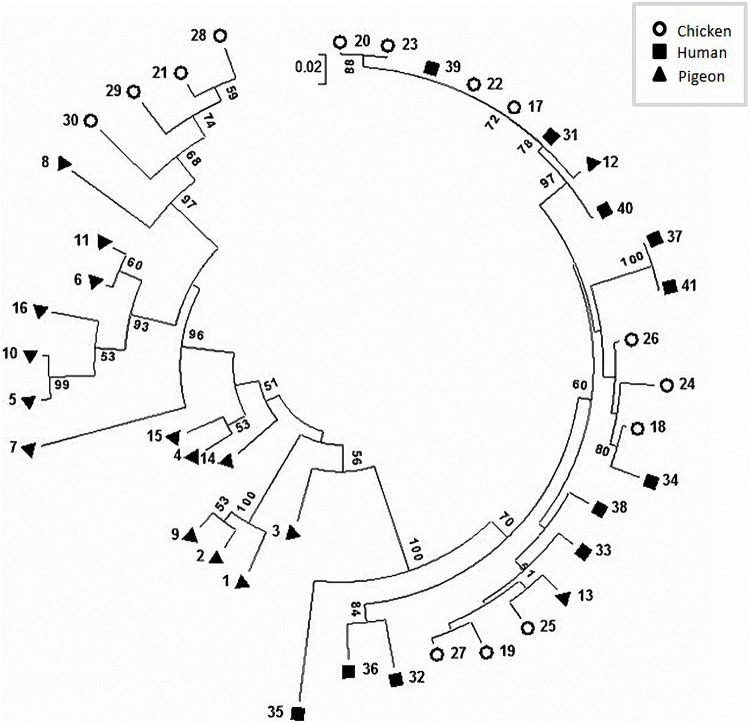
Circular phylogenetic tree of the *flaA*-SVR sequences generated using the neighbour-joining method. The numbers on each branch indicate the calculated bootstrap values. The scale-bar indicates a branch length equivalent to a 2% difference in the nucleotide sequence.

The phylogenetic analysis (Figure 4) and the proximity matrix (Supplementary Table S2) revealed sequence heterogeneity among our *C. jejuni* strains. While pigeon strains were phylogenetically related, some of them were clustered close to (e.g., strain No. 13) or shared the same lineage with (e.g., strain No. 12) chicken or human strains. The proximity matrix (Supplementary Table S2) indicated that the bacterial population within chickens and pigeons had less similarity on average (average similarity, each = 86.9%) than those within humans (average similarity = 93.4%).

### Allelic Diversity of *C. jejuni*

We identified 17 and 12 allele variants at nucleotide and peptide levels, respectively (Table 4 and Supplementary Table S1) with 13 novel alleles at the nucleotide level (Bold numbers in Supplementary Table S1 and Table 4). Irrespective of the strain source, allele 177 was the most frequently identified nucleotide variant (13/41, 31.71%), followed by allele 288 (*n* = 4, 9.76%). The *flaA*-SVR nucleotide allele 177 was the dominant one in human (6/11, 54.55%) and chicken (6/14, 42.86%) strains, whereas alleles 718, 38 and 1118 were the most frequent in pigeon strains (2/16, 12.5%, each). Certain allele variants were not identified in human and chicken strains, yet were detected in pigeon strains (Supplementary Table S1). At the peptide level, allele 74 was the most frequently identified variant (13/41, 31.71%), followed by allele 92 (7/41, 17.07%) and allele 239 (4/41, 9.76%). The *flaA*-SVR peptide allele 74 was the most prevalent in human (6/11, 54.55%) and chicken (6/14, 42.86%) strains, whereas allele 239 predominated in pigeon strains (4/16, 25%). The nMDS plots that was built based on all identified alleles (Supplementary Figures S2A,B) indicated that *C. jejuni* strains isolated from humans and birds were highly diverse and overlapped largely. Considering all identified alleles (at both the nucleotide and peptide levels), human strains were clustered with chicken strains and were positioned apart from pigeon ones (Supplementary Figures S2C,D).

## Discussion

*Campylobacter jejuni* can colonise and infect domestic poultry and pose a risk for humans. In the current study, we analysed the genetic heterogeneity of Egyptian *C. jejuni* isolates recovered from avian and human sources. Characterisation of *C. jejuni* isolates from different sources is vital for a better understanding of the impact of multiple sources on the disease burden. The current study revealed low overall prevalence rates of *C. jejuni* in chicken and human samples (15.56 and 4.07%, respectively). Similar prevalence rates have been reported in a recent study conducted in Egypt (18.12 and 4.1%, respectively) (Ahmed et al., 2015). Moreover, the carriage rate of *C. jejuni* in pigeon droplets (8.89%) was found to be much lower than that in chickens. A previous Canadian study reported also a low infection rate of *C. jejuni* in pigeons (9.1%) (Gabriele-Rivet et al., 2016).

The observation that the *flaA* gene was encoded by all analysed strains suggests the importance of this gene as a virulence marker in our Egyptian *C. jejuni* strains and possibly other *C. jejuni*. Our results corporate previous reports (e.g., Datta et al., 2003; Talukder et al., 2008; Rizal et al., 2010; Wieczorek et al., 2012; Shyaka et al., 2015), which also identified *flaA* gene in 100% of *C. jejuni* strains. However, a lower prevalence of *flaA* gene (75.5%) was reported in *C. jejuni* isolated from chicken meat in Brazil (Melo et al., 2013). This difference may be attributed to the isolate source, sample type and geographical location (Corcoran et al., 2006).

The *cdtA*, *cdtB*, and *cdtC* were detected in the analysed strains by the same percentage (80.49%), and more than half (58.54%) of the strains possessed the three CDT toxin genes together. Previous studies (Datta et al., 2003; Rozynek et al., 2005) reported that the prevalence of each of *cdtA*, *cdtB*, or *cdtC* genes in *C. jejuni* isolated from poultry and humans exceeded 80%. The high frequency of the co-existence of these subunits and their close genotypic association (Figures 2B, 3A) was previously reported in Poland (70.8%) (Wieczorek and Osek, 2011) indicating that they are highly likely to co-express during an infection event. This is supported by the knowledge that these three subunits are required for full CDT toxin activity (Martinez et al., 2006).

The random forest classification and ROC analyses applied herein showed that the *cdtC* gene exhibited the highest similarity and thus the least discriminatory power (low AUC values) among *C. jejuni* strains from all examined sources, whereas *iam* and *cdtB* were the highest dissimilar genes among strains from different sources and that *iam* gene has relatively high discriminatory power (high AUC value) than *cdtC* gene. It is plausible to assume that the genes (e.g., *cdtC*), which have similar frequency and low discriminatory power among strains isolated from different hosts could infer about the potential sources of infection if their expression potential was tracked back after an outbreak. However, the genes (e.g., *iam* and *cdtB*), which tend to have dissimilar frequencies and have high discriminatory power could differentiate among infection sources. While this result was based on a small data set, it suggests the potential importance of certain genes in tracking and differentiating the sources of *C. jejuni* infection in human in small outbreaks. An additional wide scale profiling that includes more virulence genes in a large number of strains is needed to generalise these data.

In the current study, we analysed both virulence gene patterns and *flaA*-SVR sequences and alleles to better understand the extent of genetic diversity of *C. jejuni* strains among and within each host. Irrespective of the strain sources, we found a clear overlap among human and bird strains. In support of this, the overall Simpson’s and Shannon’s diversity indices were relatively high (Table 3). There was also a random distribution of the virulence gene patterns across the strains. Similar variabilities in the prevalence of virulence genes among *C. jejuni* have been reported previously (Abu-Madi et al., 2016). Taken together, these data reflect the high genetic diversity of our Egyptian *C. jejuni* strains.

Sequence-based *flaA*-SVR typing has been reported as a reliable genotyping method yielding reproducible results (Acke et al., 2010; El-Adawy et al., 2013). In the current study, the distribution of *flaA*-SVR alleles highlighted the diversity of the studied Egyptian *C. jejuni* strains when compared to those described previously in the PubMLST database. The variability in *flaA-*SVR gene sequences and alleles (Supplementary Figure S2) supported the impression from the virulence gene heterogeneity and further indicated the genotypic diversity of *C. jejuni*. The observed variation in the alleles/peptides might be due to the occurrence of distinct mutations (e.g., transition, transversion, additions or deletions). These genetic alterations could afford a survival advantage of the bacteria, which in turn might lead to evasion of the host immune response (Jerome et al., 2011). A similar heterogeneity was also obtained by Corcoran et al. who studied 41 *C. jejuni* strains from humans and poultry (Corcoran et al., 2006). Moreover, Ramees et al. (2015) reported a diversity of 65% among 32 analysed *C. jejuni* strains irrespective of their sources.

The current analysis showed that *C. jejuni* strains isolated from the same host did not reveal a similar frequency or distribution of both virulence genes and the *flaA* allelic variants (Supplementary Table S1), suggesting that *C. jejuni* strains studied here are not host-specific. Similar diversity was also shown previously (Corcoran et al., 2006) after analysing *flaA* gene of *C. jejuni* isolated from humans and poultry. Moreover, Vidal et al. (2016) reported a high genotypic diversity of *C. jejuni* isolated from a chicken broiler flock. The particular distribution of certain strains in certain niche could be attributed to differential response to environmental factors and/or management practices. This, in turn, will modulate the bacterial phenotypic properties (e.g., infectivity, survival and pathogenicity) and ultimately the colonisation in the host (Ahmed et al., 2002; Vidal et al., 2016). Taken together, these results indicate the existence of *C. jejuni* as quasi-species colonising the same host, which demonstrates the low host adaptability and the weak clonality of these *C. jejuni* strains as was suggested previously (Corcoran et al., 2006).

As evidenced by the measurement of diversity indices, virulence gene patterns and the average similarity of *flaA*-SVR sequences, chicken strains were found to be highly diverse relative to those from human or pigeon. The rationale behind the high diversity of *C. jejuni* in this particular host is not yet clear. However, the uniqueness of the chicken as a host for such bacteria (Awad et al., 2018) and the high load of *C. jejuni* (10^6^ – 10^8^ colony forming units/g) (Beery et al., 1988) in chicken gut might account for this heterogeneity, which usually stems from frequent recombination and plasmid transfer among strains in the same niche. These data demonstrate the importance of chickens in the epidemiology and infection dynamics of *C. jejuni* in Egypt.

Applying a hierarchical clustering on the frequency of the presence of each virulence gene (Figure 3A) and allelic/peptide variants (Supplementary Figures S2C,D) in *C. jejuni* strains revealed that human strains were genotypically clustered close to chicken strains rather than to pigeon ones. While this needs to be interpreted using large studies, it suggests the importance of chicken as a source of human infection with *C. jejuni* as was stated previously (Oh et al., 2017).

A shortcoming of this study is due to the limited funding resources. Therefore, we described the diversity of *C. jejuni* depending on sequence-based typing of *flaA-*SVR gene, which is less costly and thus fits more the situation in developing countries, notably Egypt. While this approach is convenient for small-scale investigations, using advanced techniques such as pulsed-field gel electrophoresis (PFGE) and MLST would have added more sensitivity and accuracy to the results and would allow comparing different typing methods at once. Future studies are warranted in this direction. In conclusion, this is the first report in Egypt that describes the extent of genetic diversity of *C. jejuni* isolated from chickens, pigeons and humans. It suggests the possible role of poultry, particularly chickens, in the transmission of *C. jejuni* to humans. It is recommended to consider these observations in the future epidemiological evaluation and risk assessment of *C. jejuni* not only in poultry, but also in humans.

## Data Availability Statement

The datasets generated for this study can be found in the GenBank database with accession numbers KX066127–KX066135, MG677923–MG677934, and MK281494–MK281513.

## Ethics Statement

The animal study was approved by the committee of Animal Welfare and Research Ethics of Faculty of Veterinary Medicine, Zagazig University. The study involving human participants was reviewed and approved by the research ethical committee of Faculty of Medicine, Zagazig University. The patients/participants provided their written informed consent to participate in this study.

## Author Contributions

MA, NA, and EA designed the study. MA and NA carried out the molecular analyses and participated in the data analysis. MS performed the bioinformatics and statistical analyses of the data. EE, MB, and RM conceived the study and participated in the design. MA, NA, and MS wrote the initial draft of the manuscript. All authors approved the final manuscript.

## Conflict of Interest

The authors declare that the research was conducted in the absence of any commercial or financial relationships that could be construed as a potential conflict of interest.

## Footnotes

1 www.ncbi.nlm.nih.gov/BLAST/

2 http://pubmlst.org/campylobacter/

## Supplementary Material

FIGURE S1 Degree of similarity of analysed virulence genes across the sources of *C. jejuni* strains. The figures show random forest classification with the predictive accuracy of each of the studied genes plotted on the *X*-axis against the respective gene name on the *Y*-axis. The mini heat maps show the frequency distribution of each gene in the respective strain sources.

FIGURE S2 Clustering patterns and diversity of *C. jejuni* strains based on frequency of *flaA*-SVR alleles. **(A,B)** Show a non-metric multidimensional scaling of the 41 analysed strains based on the presence and absence of all alleles of the *flaA*-SVR nucleotides **(A)** and proteins **(B)**. **(C,D)** Show clustering pattern of *C. jejuni* strain sources (based on Euclidean distance) using the frequency of alleles at the nucleotide **(C)** and protein **(D)** levels as input data.

TABLE S1 Frequency of virulence gene combinations, and nucleotide and peptide alleles among 41*C. jejuni* strains from different sources.

TABLE S2 Distance matrix showing the percentage of similarity between each pair of the strains based on the alignment of *flaA*-SVR sequences. HU: humans, PI: pigeon, CK: chicken. ^∗∗∗^ indicates 100% similarity.
