# Supplementary figures and images for: Genetic Diversity of Campylobacter jejuni Isolated From Avian and Human Sources in Egypt
Source: Front Microbiol. 2019 Oct 18;10:2353. doi: 10.3389/fmicb.2019.02353 (PMC6813243; doi:10.3389/fmicb.2019.02353)

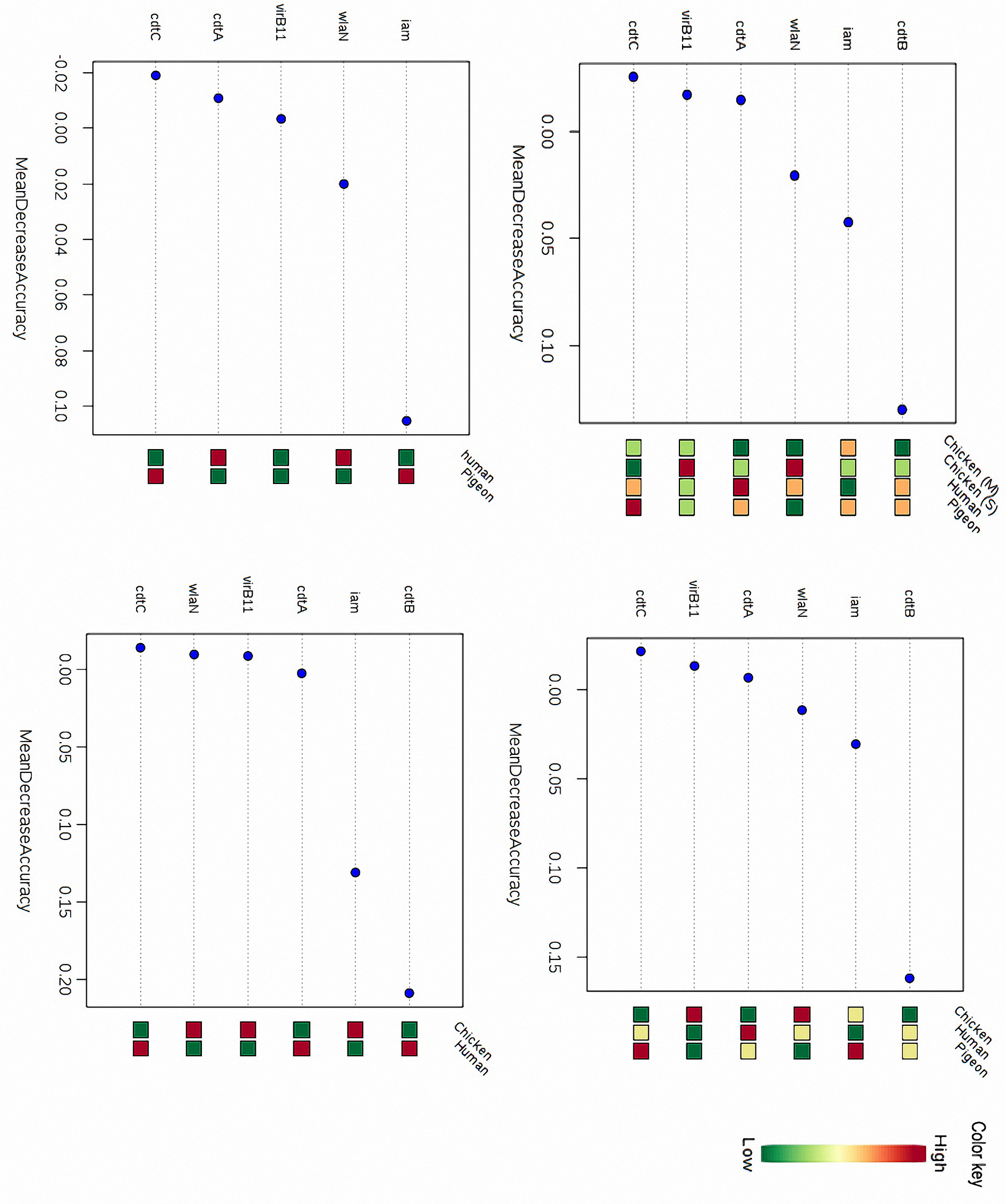

Supplement: FIGURE S1 — Degree of similarity of analysed virulence genes across the sources of C. jejuni strains. The figures show random forest classification with the predictive accuracy of each of the studied genes plotted on the X-axis against the respective gene name on the Y-axis. The mini heat maps show the frequency distribution of each gene in the respective strain sources. [file Image_1.TIF]

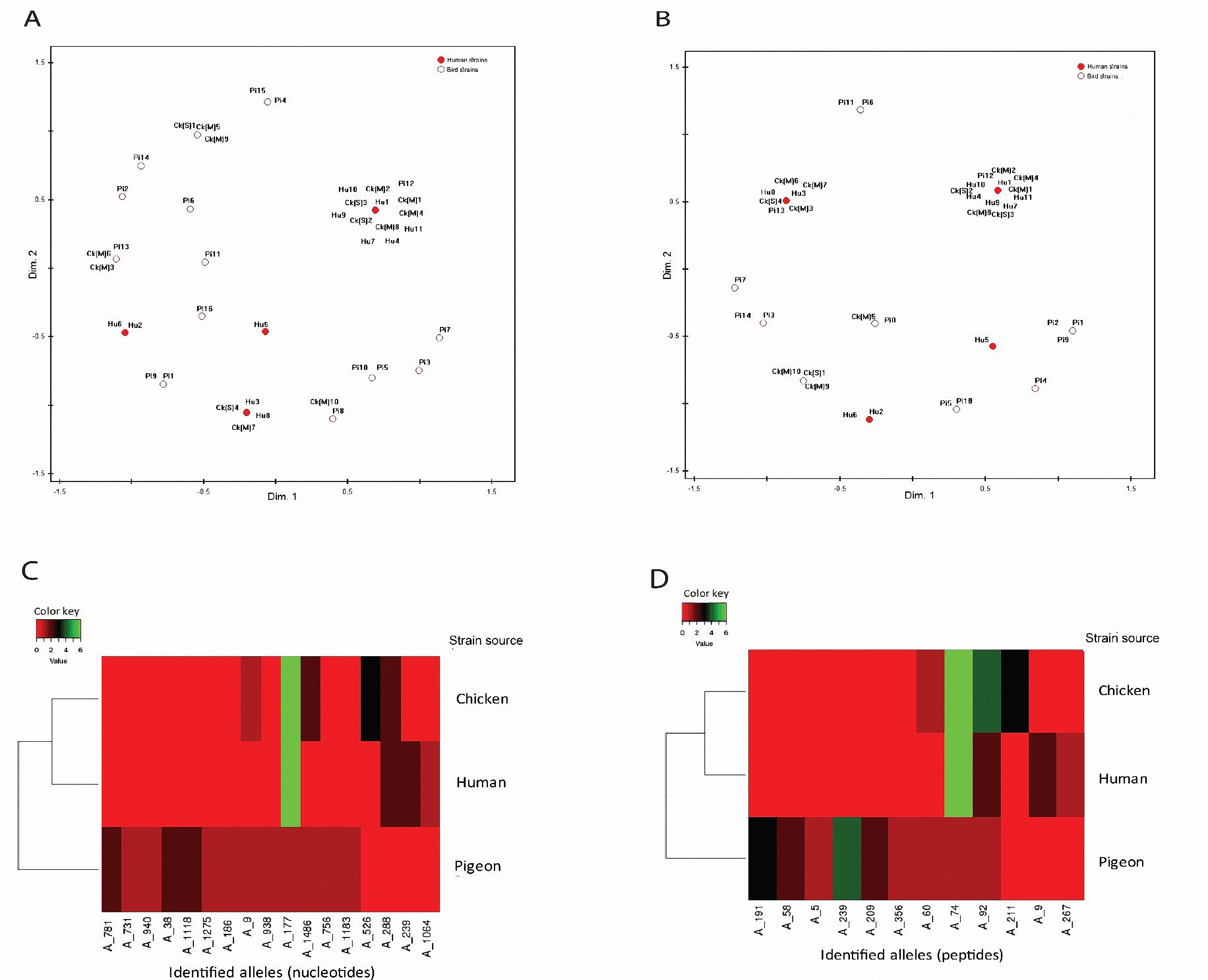

Supplement: FIGURE S2 — Clustering patterns and diversity of C. jejuni strains based on frequency of flaA-SVR alleles. (A,B) Show a non-metric multidimensional scaling of the 41 analysed strains based on the presence and absence of all alleles of the flaA-SVR nucleotides (A) and proteins (B). (C,D) Show clustering pattern of C. jejuni strain sources (based on Euclidean distance) using the frequency of alleles at the nucleotide (C) and protein (D) levels as input data. [file Image_2.TIF]
