# Supplementary material for: Genetic Diversity of Campylobacter jejuni Isolated From Avian and Human Sources in Egypt
Source: Front Microbiol. 2019 Oct 18;10:2353. doi: 10.3389/fmicb.2019.02353 (PMC6813243; doi:10.3389/fmicb.2019.02353)
Supplement: TABLE S1 — Frequency of virulence gene combinations, and nucleotide and peptide alleles among 41C. jejuni strains from different sources. [file Table_1.DOCX]

**Table S1**. Frequency of virulence gene combinations, and nucleotide and peptide alleles among 41*C. jejuni* strains from different sources

| **source**  **Criteria** | **Human**  **(n=11)** | **Pigeon**  **(n=16)** | **Chicken**  **(n=14)** | **All sources**  **(n=41)** |
| --- | --- | --- | --- | --- |
| Gene profile |  | | | |
| *cdtABC*, *flaA*, *virB11*, *iam* | 0 | 1 | 1 | 2 |
| *cdtABC*, *flaA*, *iam* | 0 | 6 | 1 | 7 |
| *cdtABC*, *flaA* | 5 | 5 | 0 | 10 |
| *cdtAB*, *flaA* | 2 | 1 | 1 | 4 |
| *cdtAB, flaA, iam* | 0 | 1 | 1 | 2 |
| *cdtBC, flaA* | 1 | 2 | 0 | 3 |
| *cdtABC, flaA, wlaN, iam* | 0 | 0 | 1 | 1 |
| *cdtABC, flaA, virB11, wlaN* | 0 | 0 | 1 | 1 |
| *cdtAC, flaA, wlaN, iam* | 0 | 0 | 1 | 1 |
| *flaA* | 0 | 0 | 2 | 2 |
| *cdtC, flaA* | 0 | 0 | 3 | 3 |
| *cdtAC, flaA, iam* | 0 | 0 | 1 | 1 |
| *cdtAC, flaA* | 0 | 0 | 1 | 1 |
| *cdtABC, flaA, virB11* | 1 | 0 | 0 | 1 |
| *cdtABC, flaA, wlaN* | 2 | 0 | 0 | 2 |
| Allele type (nucleotides) | | | | |
| **781** | 0 | 2 | 0 | 2 |
| **731** | 0 | 1 | 0 | 1 |
| **940** | 0 | 1 | 0 | 1 |
| 38 | 0 | 2 | 0 | 2 |
| **1118** | 0 | 2 | 0 | 2 |
| **1275** | 0 | 1 | 0 | 1 |
| **186** | 0 | 1 | 0 | 1 |
| 9 | 0 | 1 | 1 | 2 |
| **938** | 0 | 1 | 0 | 1 |
| 177 | 6 | 1 | 6 | 13 |
| **1486** | 0 | 1 | 2 | 3 |
| **756** | 0 | 1 | 0 | 1 |
| **1183** | 0 | 1 | 0 | 1 |
| **526** | 0 | 0 | 3 | 3 |
| **288** | 2 | 0 | 2 | 4 |
| 239 | 2 | 0 | 0 | 2 |
| **1064** | 1 | 0 | 0 | 1 |
| Allele type (protein) | | | | |
| 191 | 0 | 3 | 0 | 3 |
| 58 | 0 | 2 | 0 | 2 |
| 5 | 0 | 1 | 0 | 1 |
| 239 | 0 | 4 | 0 | 4 |
| 209 | 0 | 2 | 0 | 2 |
| 356 | 0 | 1 | 0 | 1 |
| 60 | 0 | 1 | 1 | 2 |
| 74 | 6 | 1 | 6 | 13 |
| 92 | 2 | 1 | 4 | 7 |
| 211 | 0 | 0 | 3 | 3 |
| 9 | 2 | 0 | 0 | 2 |
| 267 | 1 | 0 | 0 | 1 |

Bold nucleotide alleles are considered novel.
